# Three Articles

**Published:** 1857-12

**Authors:** 


### THREE ARTICLES.

In turning over the pages of this Journal, three articles attracted our attention, which we had altogether forgotten. The first, for clergymen; the second, for parents; the third, for all, as of the highest practical value—their lessons are even terrible: *Tobacco, its Use and End*, page 100; *The Children of the Rich*, page 123, first volume; *Annual Ailments*, page 145; also page 249.

